# Attention and spatial navigation in everyday life: Physical activity is associated with subjective aspects of cognitive function

**DOI:** 10.1371/journal.pone.0321062

**Published:** 2025-04-16

**Authors:** G. Kyle Gooderham, Todd C. Handy

**Affiliations:** Psychology, University of British Columbia, Vancouver, British Columbia, Canada; University of Valencia: Universitat de Valencia, SPAIN

## Abstract

Efforts to understand the effects of physical activity on cognitive health have long relied on employing objective measures that assess the efficacy of the mechanics of cognition. However, this perspective overlooks complementary dimensions of cognitive functioning, namely one’s subjective appraisal of the efficacy of their cognitive mechanics. In a set of four investigations (N =  2965), we sought to discern whether physical activity (PA), and other health and demographic factors, contribute to subjective experiences of cognitive mechanics (SCF) and to map for future investigations domains of function that are sensitive to health factors. We employed linear multiple regression analyses to examine survey data collected online from four large samples of young adults who responded to measures of health behaviours and SCF. PA contributed to subjective experiences of attentional control and spatial navigation but not memory, executive function, or general cognitive functioning. Further, sleep, diet, and stress were each consistently associated with selective measures of subjective experiences of cognition. Taken together, these studies indicate the importance of PA, as well as additional health behaviours, as significant contributors to SCF.

## Introduction

Physical activity (PA) has profound effects on the body and the brain. Nearly a century’s worth of research, dating back to at least the 1930’s [1], has repeatedly shown that participating in PA positively impacts cognitive function [2]. For example, executive control, attention, and memory are all improved by long-term [3] and short-term PA interventions [4,5]. In terms of underlying neurophysiological mechanisms, PA has been linked to increased circulation of growth factors which promote neurogenesis, synaptogenesis, dendritic growth, and angiogenesis [6–8]. Importantly, these cognitive and neurophysiological effects are most impactful at the two ends of the lifespan trajectory –– in the young, developing brain, and the older, aging brain. However, despite similar apparent impacts of PA at a neurophysiological level in young, healthy adults [9], there appear to be limited or minimal corresponding impacts at the cognitive level [10]. Such evidence, or lack thereof, has thus led to the claim that in young adults PA levels have no systematic influence on the efficacy of cognitive function [11].

From one perspective such conclusions are perhaps not surprising. In general, the physiological integrity of our neurocognitive systems is indeed at its peak in young adulthood [12,13], and quite simply, cognitive capacities in this age group are likely to operate at or near ceiling levels in terms of their functional potential [14]. If so, while PA may exert a general positive effect on neurophysiological systems associated with cognition even in young adults, such effects may only be observable in cognitive measures when baseline cognitive function is sufficiently below its maximum potential in a given individual and/or population, such as in the case of the aging brain [12,15,16]. This we label here *the ceiling hypothesis*.

On the other hand, a complementary perspective is that PA may in fact exert a positive influence on cognitive function even in young adults, but in ways that have remained relatively understudied in the PA-cognition literature. In particular, cognition itself can be understood as consisting of two elementary components –– *cognitive mechanics*, or those genetically-specified systems in the brain that support cognition as a set of neurobiologically-implemented processes, and *cognitive pragmatics*, or the experience- and culture-based knowledge and information on which our cognitive mechanics operate [17]. While we do not have any specific hypotheses regarding how PA levels may impact cognitive pragmatics, it stands to reason that there are at least three dissociable ways in which PA levels may influence cognitive mechanics –– (1) in terms of the functional efficacy of one’s mechanics per se (or one’s objectively-measurable cognitive performance), (2) in terms of the subjective experience of one’s on-going mechanics (or how one feels about their cognitive performance at any moment; [18]), and (3) in terms of the meta-level processes that guide and inform the on-going, goal-directed use of our mechanics (or the information and processes we use to monitor our cognitive performance in real-time and exert strategic control over our cognitive resources; [19]).

Given this conceptual framing of cognitive mechanics and its three dissociable elements, our study here was predicated on the assumption that the *ceiling hypothesis* specifically applies to the functional efficacy of cognitive mechanics. That is, we suggest that the bulk of evidence supporting the conclusion that PA levels do not impact cognition in young adults has come from studies using measures that most closely align with assessing the efficacy of cognitive mechanics per se, such as Stroop and other executive function tasks. As such, whether PA levels in young adults may in fact influence the subjective experience of their cognitive function (SCF) and/or their meta-cognitive monitoring and control remains a more open question. Here we specifically address the potential relationship between PA and SCF, while in our companion paper [20] we address the relationship between PA and metacognitive function.

SCF can be defined as the subjective appraisal of one’s own cognitive function at any moment, and can be positively or negatively valanced, such that when prompted one can report enhanced or impaired cognitive function relative to one’s own sense of a normative/baseline cognitive capacity [21]. For example, one might feel at a cognitive peak the morning after a night of good, high-quality sleep, while conversely, one might feel at a cognitive low the morning after a hard night of drinking, and disrupted, low-quality sleep. Measures of SCF are designed to capture such ebbs and flows in our own on-going cognitive experience.

From a health and wellbeing perspective, the question of whether PA levels impact SCF in young adult populations is arguably a vital one. Research examining the relationship between PA and cognition implicitly –– if not explicitly –– assumes that one’s health and wellbeing positively scales with the functional efficacy of one’s cognitive mechanics. While this may well be true, it’s no less reasonable to posit that how one feels about their on-going cognitive function or performance also has a direct impact on our health and wellbeing. That is, consistent with the growing awareness of mindfulness as a beneficial mental health practice, our sense of wellbeing flows not just from the objective efficacy of our neurocognitive function, but our subjective experiences of that function. In turn, studies have highlighted the potential for PA to affect SCF in middle aged and older adults [22,23] as well as in clinical populations [24–26], yet whether a similar relationship extends to young adults remains uncertain.

On the one hand, there has been some initial preliminary support for a relationship between PA and SCF in young adults. For example, Fitzsimmons et al. [27] report that daily variability in self-reported PA and sedentary behaviours are correlated with concurrent fluctuations in subjective experiences of cognitive function. In this study, 128 university students between the ages of 18 and 25 self-reported daily PA and sedentary behaviour as well as perceived cognitive ability for 14 consecutive days. Analysis revealed that participants reported greater subjective cognitive abilities on days when they self-reported greater PA or lower levels of sedentary behaviour than their average. Critically, neither PA nor sedentary behaviours averaged over the 14 days, subjectively or objectively assessed, were correlated with perceived cognitive ability. Likewise, in a sample of 1767 Brazilian adults (*M* age =  38.2), Feter et al. [28] investigated the relationship between subjective memory impairment and participation in PA both before and after the implementation of COVID-19 social distancing measures. The authors report a direct effect of physical inactivity during the lockdown on the likelihood of reporting subjective memory complaints. Furthermore, a comparison of changes to PA and subjective memory before and during social distancing revealed that individuals who became or remained more physically active were less likely to report subjective memory impairments while those who became or remained less physically activity were more likely to report subjective memory impairments.

On the other hand, other studies in young adults have found no such links. For example, no significant relationship between PA and subjective prospective or retrospective memory was observed in a recent sample of university students [29]. Additionally, in an analysis of data from the World Health Survey, Felez-Nobrega et al. [30] examined the relationship between PA and subjective cognitive impairment in 47 low and middle income nations. Despite a significant effect of PA on SCF for adults 45 years and older and a significant overall effect for the combined age groups (i.e., 18-65 + years), no significant association between low engagement in PA and either memory, concentration, or learning a new task performance was observed in younger adults aged 18-44 years. Taken together, these studies indicate that PA may have consequences for SCF in young adults, though the extent of these effects is not well documented.

Our study here was designed to extend research on the association between PA and SCF in young adults in two critical ways. First, one aim was to broaden the range of SCFs under investigation in a single study, on the hypothesis that there may be variability in what specific domains of SCF may be labile to modulation by PA. Second, both sleep and diet are LSBs associated with cognitive function. For example, disruptions to sleep patterns have considerable deleterious effects on psychological wellbeing and cognitive function [31], and similarly, poor dietary habits may also contribute to cognitive impairment [32]. In adult populations, PA, diet, and sleep cluster, such that there is high co-occurrence of healthy, or unhealthy, LSBs [33], and combined, these LSBs are positively associated with psychological wellbeing in young adults [34]. Accordingly, the second aim of our study was to control for these two LSBs as possible moderating influences on the relationship between PA and SCF in young adults. we hypothesized that better sleep habits, healthier diets, and greater cumulative PA would be associated with higher subjective ratings of cognitive performance.

## Study One

The purpose of this study was to generate insight into dimensions of SCF that may be susceptible to the health factors of PA, diet, and sleep. To do so, we recruited four samples of young adults to complete an online survey about their health behaviours and SCF. We operationalized the variables of interest with previously validated measures, with the health and demographic factors serving as predictor variables and SCF as outcome variables in a series of linear multiple regression analyses. Descriptive statistics of the participant demographics and predictor variables for each study, as well as an indicator of the internal consistency of the response variables (Cronbach’s alphas), are available in Table 1.

**Table 1:** Descriptive statistics for studies one-four.

	Study One				Study Two				Study Three				Study Four			
	*n*	*M*	*SD*	α	*n*	*M*	*SD*	*α*	*n*	*M*	*SD*	α	*n*	*M*	*SD*	*α*
Predictor Variables																
Age	998	20.78	2.44		913	20.68	2.40		506	20.67	2.02		548	20.70	2.26	
Sex	998				913				506				548			
Female	737				684				359				411			
Male	261				229				147				137			
IPAQ	912	2851.01	2713.48		837	2595.76	2371.76		464	3440.12	3022.89		510	3528.30	3098.38	
PSS	970	21.83	6.40		867	22.26	6.52		505	21.57	6.31		507	21.36	6.30	
PSQI	959	7.37	3.43		855	7.37	3.43		501	7.34	3.26		511	7.33	3.26	
REAP-S	967	30.08	4.37		866	30.06	4.29		504	30.17	4.41		491	29.52	4.68	
Response Variables																
AEFI																
Attention	951	6.64	1.73	.81												
Planning and Initiative	952	5.44	1.73	.73												
Self-Control and Self-Monitoring	956	6.30	1.95	.69												
CFQ	947	42.42	15.91	.93												
SAM																
Episodic	950	98.14	13.27	.81									499	97.90	12.82	.80
Semantic	950	96.38	11.23	.58									499	95.44	11.08	.60
Spatial	950	91.64	15.04	.76									500	89.98	14.33	.74
Future	950	94.99	14.18	.88									499	92.86	12.97	.87
ACS					801	48.36	7.70	.81	455	48.68	7.72	.80	477	48.88	7.52	.79
Focusing					834	17.61	4.07	.83	480	17.39	4.18	.84	497	17.67	4.09	.83
Shifting					837	11.43	2.58	.73	478	11.82	2.60	.72	497	11.66	2.52	.71
MWQ					779	18.51	4.67	.87	439	18.79	4.61	.88	455	18.51	4.34	.85
PRMQ					810	40.57	10.11	.91								
Prospective					828	20.97	5.50	.85								
Retrospective					822	19.59	5.17	.82								
Long-term					822	19.70	5.31	.82								
Short-term					827	20.86	5.27	.85								
Environment-cued					819	19.76	5.14	.83								
Self-cued					829	20.80	5.42	.83								
SBSOD													487	58.97	16.36	.90

## Materials and methods

1084 participants were recruited from the University of British Columbia Department of Psychology’s Human Subject Pool and remunerated with course credit. Participants provided written informed consent prior to data collection. 86 responses were removed from the dataset as the participants did not consent to the study, did not provide demographic information included in the analyses, or were identified as outliers, leaving a sample of 998 participants. The sample was drawn from students enrolled in the Fall 2020 Human Subject Pool data collection period, running 1 September 2020 until 31 December 2020. The University of British Columbia Behavioural Research Ethics Board provided ethical approval for the study program, certificate H19-02890.

### Materials

#### Participant demographics.

Respondents completed a demographic battery indicating, amongst other items, their age and sex.

#### International physical activity questionnaire.

The International Physical Activity Questionnaire (IPAQ) is a self-reported measure of PA. Respondents are prompted to report their PA during the past seven days across occupational, transportation, domestic, and leisure domains. The scoring protocol provides for the calculation of Metabolic Equivalent of Task (MET) values corresponding to the exertion required to complete the task [35]. The IPAQ has high validity and reliability [36,37], and has been demonstrated to be stable and accurate in young adult populations [38].

#### Perceived stress scale 10.

The Perceived Stress Scale 10 (PSS) is a revised version of Cohen et al’s. [39] self-report measure of appraised stress [40]. Respondents are asked to indicate the frequency with which they felt or thought a certain way in response to ten life stressors, with high scores indicating greater degrees of perceived stress. The PSS 10 is a widely used measure of stress and, despite concerns over the number and organization of factors [41], is found to have satisfactory reliability and convergent validity with more comprehensive measures of stress in young adults [42].

#### Pittsburgh sleep quality index.

The Pittsburgh Sleep Quality Index (PSQI) is a self-reported measure of sleep quality in the preceding month [43]. Participants are asked to provide information pertaining to sleep efficiency, trouble sleeping, and interference on other tasks because of poor sleep. From these questions a composite score is computed, with higher scores indicating worse sleep quality. The PSQI has demonstrated good reliability and validity in a meta-analysis of thirty-seven studies of clinical and non-clinical samples [44].

#### Rapid eating and activity assessment for participants short version.

The Rapid Eating and Activity Assessment for Participants Short Version (REAP-S) is a sixteen-item self-report measure of an individual’s dietary habits [45]. Respondents are prompted to indicate the frequency of achieving certain dietary thresholds, such as meeting daily consumption of servings of fruit and vegetables, in an average week. Thirteen of the items are scored on a likert scale with response options of Does not apply to me, Rarely/Never, Sometimes, and Usually/Often, corresponding to scores of 3, 3, 2, and 1. The scores on these items are summed, with higher scores indicating healthier dietary habits. The REAP-S has shown good validity when compared with more comprehensive measures of dietary habits in young adults [45] in addition to a positive correlation with chemical indices of diet quality [46].

#### Amsterdam executive function index 10.

The Amsterdam Executive Function Index 10 (AEFI) is a self-reported measure of attention, planning, and self-control and self-monitoring [47]. The ten-item scale probes respondents with a three-response likert scale, with higher scores signalling poorer executive function. The original AEFI demonstrated satisfactory validity and reliability [48], though the revised ten-item version was shown to have better reliability than the original thirteen-item index [47].

#### Cognitive failures questionnaire.

The Cognitive Failures Questionnaire (CFQ) is a twenty-five item self-reported measure of failures of perception, memory, and motor function [49]. As an indices of trait level cognitive function, the CFQ has been shown to sufficient reliability and validity [50].

#### Survey of autobiographical memory.

The Survey of Autobiographical Memory (SAM) is a self-reported measure of an individual’s perceived trait level memory performance [51]. Episodic, semantic, spatial, and future prospective memory function are assessed across the twenty-six-item questionnaire. Psychometric evaluation of the SAM has demonstrated the measure to have satisfactory reliability across each component of memory as well as the entire scale [52]. A sample of young adults demonstrated a similar pattern of reliability across the measure, though introduced concerns regarding SAM and its associations with behavioural and other self-report measures of memory performance [53].

### Methods

The entire study was done on-line. Upon enrolling in the study through the Human Subject Pool (HSP) system, participants were provided with a link through the HSP system to the survey, distributed with the online survey tool Qualtrics. The survey was compatible with both desktop and mobile platforms and was completed on the participant’s own personal device(s). After providing informed consent as the first step, participants then completed a series of demographic questions followed by survey blocks of each of the (1) predictor and (2) response variables, presented in random, intermixed order.

All data analysis, including measure scoring, cleaning, and outlier identification and rejection, was conducted in R. Quantification of each measure followed the scoring protocol provided in the manuscripts describing the measure. Outlying data points on the predictor variables were detected using Tukey’s fences method. With this methodology, observations exceeding 1.5 times the inter-quartile range below Q1 or above Q3 were identified as outliers and removed from the dataset. Post hoc longstring analysis was performed on the response variables to identify careless responses. Using this technique, respondent data was removed from the data set if they provided the same consecutive response option to more than 90% of the measure length. For example, the CFQ consists of 25 questions, therefore if a respondent provided the same response for more than 22.5 consecutive questions they were removed from further analysis. Observations were removed based on missingness of variable scores from individual analyses.

All regression models used participant’s age, sex, perceived stress, dietary habits, sleep quality, and PA as predictor variables. Subscales of the AEFI, CFQ, and SAM served as the response variable in each model.

## Results

Variance inflation factors were calculated for each model due to the potential for collinearity between predictors within the regression models. All variance inflation factors were within the acceptable range (<1.5).

PA was significantly predictive of the AEFI Planning and Initiative subscale, such that participants who reported more PA self-reported better behavioural planning and initiative, *β* =  -0.10, 95% CI [-0.17, -0.04], *t*(847) =  -3.21, *p* = .001. Additionally, PA was marginally significantly associated with spatial ability, as assessed by the SAM Spatial Ability subscale, *β* =  0.06, 95% CI [-0.002, 0.13], *t*(848) =  1.90, *p* = .06. None of the other subscales on the AEFI, CFQ, or SAM were associated with PA.

Analysis revealed consistent significant relationships between participant diet and sleep habits with the CFQ and subscales of the AEFI such that more healthful behaviours were associated with better executive functioning. However, neither diet nor sleep habits were associated with any of the subscales of the SAM (see Table 2).

**Table 2:** Beta weights and model fit for Study One.

		AEFI						CFQ		SAM						
		Attention		Planning		Self-Control and Self-Monitoring				Episodic		Semantic		Spatial		Future
Intercept	*β*	-.01		-.08	^*^	.01		.04		.01		.01		-.15	^***^	.03
Age	*β*	-.07	*	-.02		-.08	^*^	-.03		-.02		-.08	^*^	.06		-.05
Sex	*β*	.08		0.363	^***^	-.10		-.17	^*^	-.06		-.01		.55	^***^	-.04
IPAQ	*β*	-.02		-.11	^**^	.02		.04		.03		-.04		.06		.02
PSS	*β*	.36	***	.25	^***^	.11	^**^	.34	^***^	-.19	^***^	-.13	^**^	-.14	^***^	-.04
PSQI	*β*	.14	**	.05		.04		.17	^***^	-.03		-.05		.01		.03
REAP-S	*β*	-.09	^**^	-.11	^**^	-.15	^***^	-.08	^*^	.01		.04		.05		-.07
Model Fit	*RSE*	0.88		0.94		0.98		0.87		0.98		1.00		0.95		1.01
	*R* ^2^	.22		.12		.06		.25		.05		.04		.11		.01
	*F*	39.83		19.42		8.71		45.92		6.61		5.11		17.40		1.12
	*p*	.000		.000		.000		.000		.000		.000		.000		.35
	*N*	853		854		857		849		855		855		855		855

*Note*.

*  *p* < .05.

** *p* < .01.

*** *p* < .001

Participant demographics were observed to be significantly associated with constructs of the response variables. In addition, perceived stress was significantly predictive of each observed measure except for SAM Future Prospective Memory.

Apart from SAM Future Prospective Memory, all models explained a significant proportion of variance. See Table 2 for beta weights and model fit.

## Study Two

Study One and Study Two were conducted concurrently but investigated different measures of SCF and recruited different participant samples. This was done to achieve three primary aims. First, Study Two broadened the types of subjective cognitive measures under examination by including two measures of attention and one additional measure of memory. Second, including a second measure of subjective memory function served as a pseudo-replication of Study One. Finally, due to the length of the included measures, we sought to reduce participant fatigue by distributing the assessments across two studies.

### Materials and methods

A total of 973 participants were recruited from the University of British Columbia Department of Psychology’s Human Subject Pool and were compensated with course credit. Participants provided written informed consent prior to data collection. 913 respondents were retained after removing those who did not consent to the study, did not provide demographic information included in the analyses, or were identified as outliers. The sample was recruited from the Fall 2020 Human Subject Pool data collection period, running 1 September 2020 until 31 December 2020. The University of British Columbia Behavioural Research Ethics Board provided ethical approval for the study program, certificate H19-02890.

### Materials

Study Two utilized the same predictor variables as Study One

#### Attentional control scale.

The Attentional Control Scale (ACS) is a self-reported measure of trait level attentional control [54]. The twenty-item measure probes respondents on the frequency with which they experience distractibility and challenges related to attentional engagement with a task. High scores are indicative of better self-perceived attentional control. Psychometric evaluation of the scale has shown the ACS to have two factors: shifting and focusing, as well as satisfactory validity in young adult populations [55].

#### Mind wandering questionnaire.

The Mind Wandering Questionnaire (MWQ) is a brief self-reported assessment of an individual’s trait level propensity to engage in task-unrelated thought [56]. The five-item questionnaire asks respondents to indicate the frequency in which they engage in mind wandering on a six-point likert scale, with larger scores corresponding to more frequent task-unrelated thought. The MWQ demonstrates sufficient validity and reliability in young adults [56].

#### Prospective and retrospective memory questionnaire.

The Prospective and Retrospective Memory Questionnaire (PRMQ) is a self-reported measure of memory failures [57]. The sixteen-item questionnaire uses a five-response likert scale, with larger scores signalling worse self-rated memory function. Respondents indicated the frequency with which they experience failures of memory in three dichotomies: prospective vs. retrospective, short vs. long-term, and self vs. environmentally cued. Confirmatory factor analysis suggests that a tripartite structure, with a general memory factor and orthogonal factors of prospective and retrospective memory, exhibited good model fit and reliability [58].

### Methods

Study Two followed the methodological implementation described for Study One

## Results

Variance inflation factors were calculated for each model due to the potential for collinearity between predictors within the regression models. All variance inflation factors were within the acceptable range (<1.5).

Analysis identified two significant relationships between PA and attentional control. First, PA and the MWQ were significantly related, to the extent that as PA increased, self-reported mind wandering decreased, *β* =  -0.09, 95% CI [-0.16, -0.02], *t*(693) =  -2.62, *p* = .009. Second, analysis of the ACS subscales revealed a significant relationship between PA and attentional Shifting, but not Focusing, *β* =  -0.12, 95% CI [0.04, 0.19], *t*(745) =  3.21, *p* = .001. Additionally, a marginally significant relationship was observed between PA and attentional control, such that as PA increased, participants reported greater attentional control, as measured by the ACS, *β* =  0.07, 95% CI [<-0.001, 0.14], *t*(712) =  1.96, *p* = .05. PA was no*t* significantly predictive of any aspect of memory function as quantified by the PRMQ.

Better self-reported sleep habits were significantly linked to enhanced memory performance, as measured by the PRMQ. Healthful diet was only predictive of better self-cued memory function and less mind wandering, but not of attentional control or other dimensions of memory function.

Participant age was a significant predictor of the MWQ and each subscale of the PRMQ, such that older participants reported less mind wandering and better memory performance. However, age was not predictive of scores on the ACS or either of its subscales.

All models explained a significant proportion of variance. See Table 3 for beta weights and model fit.

**Table 3:** Beta weights and model fit for Study Two.

		ACS						MWQ		PRMQ													
		ACS		Focusing		Shifting				PRMQ		Prospective		Retrospective		Short-term		Long-term		Self-cued		Environment-cued	
Intercept	*β*	-.03		.00		-.05		-.02		-.01		-.01		-.01		.00		-.01		.00		-.01	
Age	*β*	-.02		.01		.00		-.10	^**^	-.10	^**^	-.07	^*^	-.11	^**^	-.10	^**^	-.09	^*^	-.10	^**^	-.08	^*^
Sex	*β*	.00		-.06		.05		.15		-.01		-.02		.00		-.02		.01		.01		-.03	
IPAQ	*β*	.07		.03		.12	^**^	-.09	^**^	-.04		-.04		-.04		-.04		-.05		-.03		-.06	
PSS	*β*	-.37	^***^	-.39	^***^	-.17	^***^	.40	^***^	.23	^***^	.24	^***^	.20	^***^	.22	^***^	.21	^***^	.23	^***^	.20	^***^
PSQI	*β*	-.01		-.06		-.04		.08		.18	^***^	.18	^***^	.16	^***^	.16	^***^	.18	^***^	.16	^***^	.19	^***^
REAP-S	*β*	.00		.04		-.02		-.07	^*^	-.05		-.07		-.04		-.06		-.05		-.08	^*^	-.03	
Model Fit	*RSE*	0.92		0.91		0.97		0.89		0.94		0.93		0.96		0.94		0.94		0.93		0.95	
	*R* ^2^	.15		.19		.05		.21		.15		.15		.11		.13		.13		.14		.12	
	*F*	21.39		27.98		7.05		31.15		20.05		21.07		15.34		18.38		18.20		19.72		17.05	
	*p*	.000		.000		.000		.000		.000		.000		.000		.000		.000		.000		.000	
	*N*	719		749		752		700		719		735		729		734		730		735		728	

*Note*.

* *p* < .05.

** *p* < .01.

*** *p* < .001

## Study Three

Study Three sought to replicate the findings of Study Two of a significant relationship between PA and attentional control. An a priori power analysis was conducted using G * Power to determine the target sample size. Informed by data obtained in Study Two, an effect size derived from the smallest observed *R*^2^ of.05, an alpha of.05, and a desired power of.95 determined a target sample size of 424 participants. The target sample size was increased by approximately 33% owing to the anticipation that some observations would be unusable, as observed in Studies One and Two.

### Materials and methods

Participants were recruited from the University of British Columbia Department of Psychology’s Human Subject Pool and granted course credit for participation. 506 responses from 579 participants were retained after removing those who did not consent to participating, did not provide demographic information included in the analyses, or were identified as outliers. Data collection occurred during the Spring 2021 Human Subject Pool data collection period, beginning 1 January 2021 until 30 April 2021. The University of British Columbia Behavioural Research Ethics Board provided ethical approval for the study program, certificate H19-02890.

### Materials

Study Three employed the same predictor variables as Studies One and Two. The ACS and MWQ, described in Study Two, served as the response variables.

### Methods

Data collection and analysis adhered to the methodology described in Study One.

## Results

Variance inflation factors were calculated for each model due to the potential for collinearity between predictors within the regression models. All variance inflation factors were within the acceptable range (<1.5).

Analysis detected significant associations between PA and the ACS Focusing subscale, *β* =  0.10, 95% CI [0.02, 0.19], *t*(426) =  2.31, *p* = .02, as well as the ACS overall, *β* =  0.11, 95% CI [0.02, 0.20], *t*(403) =  2.34, *p* = .02, such that more physically active participants reported better attentional control. However, non-significant relationships were observed between PA and the ACS Shifting subscale and the MWQ.

Participant sleep was significantly predictive of the MWQ, ACS, and the ACS Focusing subscale, with better sleep habits linked to better attentional control. Dietary habits were not significantly related to either of the outcome measures.

Perceived stress was significantly predictive of all the response variables, with less stress associated with enhanced subjective cognitive performance. Neither participant age nor sex were significantly linked to self-reported attentional function.

All models explained a significant proportion of variance. See Table 4 for beta weights and model fit.

**Table 4:** Beta weights and model fit for Study Three.

		ACS						MWQ	
		ACS		Focusing		Shifting			
Intercept	*β*	-.02		.00		-.04		.06	
Age	*β*	.01		.06		-.02		-.07	
Sex	*β*	.05		.00		.11		-.09	
IPAQ	*β*	.11	^*^	.10	^*^	.07		-.04	
PSS	*β*	-.31	^***^	-.31	^***^	-.16	^**^	.32	^***^
PSQI	*β*	-.15	^**^	-.19	^***^	-.05		.16	^**^
REAP-S	*β*	-.03		-.03		.00		-.01	
Model Fit	*RSE*	0.93		0.89		0.98		0.88	
	*R* ^2^	.17		.20		.05		.19	
	*F*	13.85		17.80		3.49		15.74	
	*p*	.000		.000		.002		.000	
	*N*	410		433		432		401	

*Note.*

* *p* < .05.

** *p* < .01.

*** *p* < .001

## Study Four

Study Four followed sequentially from Study Three with the aim of replicating and extending the findings of a relationship between PA and spatial ability as well as PA and attentional control. An a priori power analysis conducted using G * Power, an effect size derived from the smallest observed *R*^2^ of.05, an alpha of.05, and a desired power of.95, informed Study Four’s target sample size of 424. As with Study Three, responses were collected from additional participants to account for expected rejected data.

### Materials and methods

623 participants were recruited from the University of British Columbia Department of Psychology’s Human Subject Pool and compensated with course credit. Participants provided written informed consent prior to the beginning of data collection. 548 respondents were retained after removing those who did not consent to the study, did not provide demographic information included in the analyses, or were identified as outliers. Data collection occurred during the Spring 2022 Human Subject Pool data collection period, running 1 January 2022 until 30 April 2022. The University of British Columbia Behavioural Research Ethics Board provided ethical approval for the study program, certificate H19-02890.

### Materials

Study Four utilized the same predictor variables as the previous three studies. In addition to the SAM, ACS, and MWQ, described in Studies One and Two, Study Four included the Santa Barbara Sense of Direction Scale as a response variable.

#### Santa barbara sense of direction scale.

The Santa Barbara Sense of Direction Scale (SBSOD) is a fifteen-item self-reported measure of navigational ability [59]. Respondents indicate agreement with statements pertaining to their sense of direction on a seven-point likert scale, with higher scores indicating better self-rated navigational ability. The scale exhibits good validity and reliability [59].

### Methods

Protocols for data collection and analysis, as described in Study One, were followed in Study Four.

## Results

Variance inflation factors were calculated for each model due to the potential for collinearity between predictors within the regression models. All variance inflation factors were within the acceptable range (<1.5).

Analysis revealed two sets of significant associations between PA and the response variables. First, PA was significantly associated with three subscales of the SAM: Episodic *β* =  0.13, 95% CI [0.04, 0.23], *t*(419) =  2.74, *p* = .006, Semantic *β* =  0.12, 95% CI [0.02, 0.21], *t*(419) =  2.39, *p* = .02, and Spatial *β* =  0.12, 95% CI [0.03, 0.22], *t*(420) =  2.58, *p* = .01. Participants who reported being more physically active reported enhanced memory functioning.

Second, a significant relationship was also observed between PA and the SBSOD, with more physically active participants reporting better navigational ability, *β* =  0.15, 95% CI [0.05, 0.25], *t*(409) =  2.98, *p* = .003.

However, unlike Studies Two and Three, non-significant relationships between PA and all measures of the MWQ and ACS were observed.

Diet was significantly predictive of scores on the MWQ, ACS, and the ACS Focusing subscale, with healthier diets linked to better attentional control. Similarly, sleep was significantly predictive of the MWQ and the ACS Focusing subscale, such that better sleep habits were associated with greater attentional control.

Perceived stress was significantly predictive of the SAM Semantic, SBSOD, and all dimensions of the MWQ and ACS, with greater reported stress subjectively impairing cognitive functioning. Further, sex was associated with the SAM Episodic, SAM Spatial, MWQ, and SBSOD. While males reported better spatial abilities and less mind wandering, females reported enhanced episodic memory performance. Age predicted SAM Spatial scores, with increasing age linked to greater self-reported spatial ability.

All models, excepting SAM Future Prospective Memory, explained a significant proportion of variance. See Table 5 for beta weights and model fit.

**Table 5:** Beta weights and model fit for Study Four.

		ACS						MWQ		SAM							SBSOD	
		ACS		Focusing		Shifting				Episodic		Semantic		Spatial		Future		
Intercept	*β*	-.02		-.03		-.05		.10		.02		.00		-.13	^*^	.04	-.12	^*^
Age	*β*	-.08		-.06		-.03		-.02		.01		-.07		.11	^*^	.03	.08	
Sex	*β*	-.01		-.01		.15		-.28	^*^	-.33	^**^	-.12		.36	^**^	-.20	.30	^*^
IPAQ	*β*	.06		-.01		.08		-.09		.13	^**^	.12	^*^	.12	^*^	.05	.15	^**^
PSS	*β*	-.31	^***^	-.31	^***^	-.16	^**^	.27	^***^	-.05		-.18	^**^	-.09		.06	-.15	^**^
PSQI	*β*	-.02		-.11	^*^	.10		.11	^*^	-.10		.07		-.06		.02	-.01	
REAP-S	*β*	.15	^**^	.14	^**^	.10		-.18	^***^	.03		.00		.00		.08	.00	
Model Fit	*RSE*	0.92		0.91		0.99		0.89		0.98		0.99		0.96		0.99	0.97	
	*R* ^2^	.16		.18		.05		.22		.05		.04		.09		.02	.09	
	*F*	12.45		15.11		3.85		18.07		3.51		3.20		6.58		1.39	6.38	
	*p*	.000		.000		.001		.000		.002		.004		.000		.217	.000	
	*N*	411		424		423		387		426		426		427		427	416	

*Note.*

* *p < .05.*

** *p < .01.*

*** *p < .001*

## Discussion

Efforts to understand the effects of PA on cognitive health have long relied on employing objective measures that assess the efficacy of the mechanics of cognition [10,60]. However, this perspective overlooks complementary dimensions of cognitive functioning, namely one’s subjective appraisal of the efficacy of their cognitive mechanics. In a set of four investigations (N =  2965), we sought to discern whether PA, and other health and demographic factors, contribute to subjective experiences of cognitive mechanics and to map for future investigations domains of function that are sensitive to health factors. We employed linear multiple regression analyses to examine survey data collected online from four large samples of young adults who responded to measures of health behaviours and SCF. PA contributed to subjective experiences of attentional control and spatial navigation but not memory, executive function, or general cognitive functioning. Further, sleep, diet, and stress were consistently associated with all measures of subjective experiences of cognition. Taken together, these studies indicate the importance of PA, as well as additional health behaviours, as significant contributors to SCF (see Table 6 for a summary of findings). From our analyses we draw five conclusions.

**Table 6:** Summary of results for Studies One-Four.

	Attentional Control	Executive Function	General Cognitive Function	Memory	Spatial Navigation
Age		*		*	
Sex			*		*
PA	*				*
Stress	*	*	*	*	*
Sleep	*		*	*	
Diet		^*^			
*R* ^2^	*	*	*	*	*

*Note*.

* denotes p < .05 in the majority of linear regression models of each measure.

First, engaging in PA is associated with enhanced experiences of subjective attentional control. The correlation between PA and subjective attentional control may be the reflection of bettered objective attentional control observed in young adults following PA [61,62]. However, discretion is warranted as we failed to directly replicate the pattern of significant relationships observed across any of our studies. These inconsistent observations mar our ability, and willingness, to draw a straightforward conclusion. Rather, the data suggest that, while a relationship between PA and subjective attentional control is noteworthy in some samples, additional factors moderate the strength of this relationship. Still, we interpret these data as signifying of *some* relationship.

Second, PA is linked with subjective spatial navigational ability. Analysis of the SAM Spatial subscale in Study One identified a link between PA and subjective spatial navigational ability that approached significance (*p* = .06). Replication and extension in Study Four revealed significant associations between PA and spatial memory as assessed by the SAM Spatial measure as well as the SBSOD. Detection of a relationship between PA and spatial ability in the subjective domain extends previous research demonstrating an improvement to objective measures in young [63] and older adult populations [64] and is suggestive that these mechanistic enhancements are subjectively experienced. Further research is required to establish a causal relationship between PA and subjective spatial navigational ability and to determine whether shifts in objective performance correlate with subjective experience.

Third, PA is not predictive of memory, executive function, or general measures of SCF. Despite satisfactory power, we did not observe consistent significant predictive relationships between PA and memory, executive function, or general measures of SCFs. PA may not broadly improve SCF but instead boost an individual’s perceived ability in a specific set of functions. This mirrors the impact of PA on objective measures of cognitive mechanics insofar as certain mechanistic functions are enhanced while others are seemingly unaffected (see [65] for a review).

Fourth, demographic and other health behaviours contribute to SCF. Analysis revealed that perceived stress and sleep habits were significantly predictive of executive function, attention, and memory performance, while diet was linked to executive control and some aspects of memory performance. Interestingly, these relationships were observed while holding for other health and LSBs, indicating that they independently contributed to SCF. These findings provide novel evidence that engagement in health behaviours for young adults is positively associated with self-reported perceptions of cognitive functioning and suggests that SCF are susceptible to interventions targeting health behaviours. Analysis of potential mediating and moderating relationships, and resultant outcomes on SCF, is necessary considering the interrelatedness of these factors [33].

Finally, notwithstanding the exception of the SAM Future Prospective Memory subscale, the tested regression model explained a significant proportion of variance for each of the measures of SCF. Thus, the extent to which SCF varies can be partially accounted for by the combined effect of demographic and health factors. Whether this meets the threshold for clinical significance necessitates interpretation with deference to the individual response variables of interest, the planned interventions, and the desired effect sizes. Nevertheless, we contend that the confluence of health behaviours serves an important role in the expression of SCF.

The interpretation of the results generates four considerations for future investigations.

First, while we observed several significant relationships between PA and measures of SCF, many of these results did not replicate or did so in unpredicted ways. Reconciling the pattern of observed results is challenging and necessitates consideration of four aspects of the study’s methodology. First, the studies are correlational and therefore the casual direction of the inferred relationships cannot be established. Second, the conventions of inferential testing may have contributed to our ability to explain the findings. Post hoc power analyses, however, indicated more than sufficient power to detect an effect in each study. Third, the distribution of the data, and its implications for the assumptions of linear regression modeling, could have jeopardized the analyses. For example, our PA measure was prone to nonnormality and the health behaviours themselves tend to covary [33]. Nevertheless, the metrics utilized to assess these assumptions met the required thresholds. Finally, characteristics of the study samples may have influenced our results. For instance, mean PA for Studies One and Two differed substantially from Studies Three and Four. This may be an artifact of depressed levels of PA resulting from the season and restrictions on social and group fitness gatherings in response to COVID-19 during the Fall of 2020 when data was collected for Studies One and Two, compared to those in the Spring of 2021 and 2022 when data was collected for Studies Three and Four. These potential effects should be addressed with modified and conceptual replications.

Second, we utilized self-reported quantifications of lifestyle behaviours which may be liable to deviations from objective indices. Special consideration must be paid to four specific issues faced by self-report measures of lifestyle behaviours as they pertain to cognitive effects. First, in non-diary collection methods, ambiguity about the intent and meaning of questions can lead to misrepresentation of the data [66]. This is particularly important when the parameters of the health behaviours, such as physical activity frequency, intensity, time, and type [67] or diet macro-nutrition [68], are critical determinants of the effects on cognition. Second, self-report measures may not appropriately capture some aspects of lifestyle behaviours because they privilege certain forms over those incidental to daily living [69]. For instance, carrying a bag of groceries requires the same recruitment of skeletal, muscular, and cognitive resources as carrying a weight yet, because it is an activity typical of daily living, may not be considered by the individual as physical activity and therefore may not be captured by a self-report measure. Likewise, nutrients are not consumed in isolation and, even within a relatively restricted diet, the consumption of different nutrients is diverse from meal to meal [70], impeding identification of single micro or macro nutrients as determining cognitive outcomes. Third, the temporal relationship between the lifestyle behaviours and cognitive effects is a key determinant of their effect [4,68,71], and these precise relationships are difficult to capture using self-report data. Finally, recall bias may result in an under- or over-representation of lifestyle behaviours [68,72,73]. Subsequently, the exact values of the reported lifestyle behaviours are prone to inaccuracies.

Though apprehension about the use of self-reported measures of lifestyle behaviours is warranted, the measures of physical activity, diet, and sleep utilized here are suitable for assessing broad engagement in these health behaviours [38,44,45]. Still, the use of device assisted assessments or more intensive self-report measures would allow for a depth of analysis that was not attainable in the present works. Given the limitations of each method of data collection, it would be prudent to combine self-report and objective measures, recognizing that neither is a perfect quantification of such complex constructs [74–76].

Third, we employed measures of SCF which may not necessarily correlate with objective measures of cognitive performance. Three concerns about subjective measures of cognitive function provide insight into the complexity of the construct. First, there exists systematic discrepancies between objective and subjective indices of cognitive function. Psychological and socio-demographic factors, including sex [77,78], age [79,80], affect and mental health [81,82], personality [83,84], and cognitive impairment [85], contribute to over and underestimation of cognitive performance. Second, subjective experiences of cognition are the result of interactions between the structural and functional integrity of cognitive processes. To illustrate, reports of cognitive failures are negatively correlated with age [80], but this may reflect changes in monitoring capabilities that accompany cognitive decline [86,87]. Finally, and unsurprisingly, some measures of subjective cognition exhibit greater convergence with objective measures of cognition than others [e.g., 88]. Setting aside issues of instrument validity and reliability, this may be in part due to metacognitive processes that enable conscious awareness of cognitive performance [89]. The inclusion of additional covariates in regression and structural equation models would provide further insight into the multifactorial relationship.

Nevertheless, four justifications support the use of subjective and self-reported measures of cognition when the research objective is to understand perceived cognitive abilities. First, objective and subjective measures of cognition are related in some populations and contexts [85]. Second, discrepancies between subjective and objective indices of cognitive function provide important information about cognitive status [90]. Third, subjective reports provide otherwise inaccessible insight into the conscious experience of cognitive functioning [91]. Finally, subjective cognition is an indicator of perceived cognitive status which recognizes the intrinsic value of the individual’s experience as valid and valuable [18]. SCF is inherently and inextricably reliant on an individual’s perception of their cognitive function, self-reports are the only viable method for probing an individual about their perceived cognitive function. Therefore, dismissing subjective measures of cognition because they are not objective measures is ill-advised because they are not intended to capture the same dimensions of cognition.

Lastly, though we have addressed an important knowledge gap and have provided novel insight into the role of PA, and other health behaviours, on subjective experiences of cognitive mechanics, we do not know if this relationship reflects mechanistic and metacognitive functioning. Future research that includes measures of objective, subjective, and metacognitive mechanics would allow researchers to determine whether there is a main effect or interaction of health behaviours on specific cognitive processes across cognitive components. Results from such a study would inform our understanding of the effects of health behaviours on cognition. If the associations between health behaviours and cognitive processes are uniformly impactful across cognitive mechanics, then this may be suggestive of a common explanatory mechanism. Conversely, an interaction would provide support for our posited ceiling hypothesis as well as have significant implications for the connection between objective, subjective, and metacognitive mechanics. In light of our findings, studies of this nature would hold theoretical and practical significance.

## Conclusion

Cognitive mechanics are comprised of more than measures of its efficacy or objective performance. Rather, one’s experience of its function offers an alternative perspective of a deeply human issue: that cognitive function is the interaction of the mind, body, and environment, in which external factors contribute significantly to that experience [92]. This is of particular importance for a young adult population that has not reaped the same benefits to objective cognitive function as other age groups [10]. Taken together, the set of findings here provide strong initial support for the idea that not only can we reliably report on our on-going cognitive status from a subjective perspective, but that this is a vital and perhaps under-appreciated domain of cognitive function positively influenced by our everyday lifestyle behaviours with respect to PA, diet, and sleep.
